# Elimination of Lymphatic Filariasis in The Gambia

**DOI:** 10.1371/journal.pntd.0003642

**Published:** 2015-03-18

**Authors:** Maria P. Rebollo, Sana Malang Sambou, Brent Thomas, Nana-Kwadwo Biritwum, Momodou C. Jaye, Louise Kelly-Hope, Alba Gonzalez Escalada, David H. Molyneux, Moses J. Bockarie

**Affiliations:** 1 Centre for Neglected Tropical Diseases, Department of Parasitology, Liverpool School of Tropical Medicine, Liverpool, United Kingdom; 2 Ministry of Health and Social Welfare, Banjul, The Gambia; 3 Ghana Health Service, Accra, Ghana; 4 Rey Juan Carlos University, Madrid, Spain; Washington University School of Medicine, UNITED STATES

## Abstract

**Background:**

The prevalence of *Wuchereria bancrofti*, which causes lymphatic filariasis (LF) in The Gambia was among the highest in Africa in the 1950s. However, surveys conducted in 1975 and 1976 revealed a dramatic decline in LF endemicity in the absence of mass drug administration (MDA). The decline in prevalence was partly attributed to a significant reduction in mosquito density through the widespread use of insecticidal nets. Based on findings elsewhere that vector control alone can interrupt LF, we asked the question in 2013 whether the rapid scale up in the use of insecticidal nets in The Gambia had interrupted LF transmission.

**Methodology/Principal Finding:**

We present here the results of three independently designed filariasis surveys conducted over a period of 17 years (1997–2013), and involving over 6000 subjects in 21 districts across all administrative divisions in The Gambia. An immunochromatographic (ICT) test was used to detect *W*. *bancrofti* antigen during all three surveys. In 2001, tests performed on stored samples collected between 1997 and 2000, in three divisions, failed to show positive individuals from two divisions that were previously highly endemic for LF, suggesting a decline towards extinction in some areas. Results of the second survey conducted in 2003 showed that LF was no longer endemic in 16 of 21 districts surveyed. The 2013 survey used a WHO recommended LF transmission verification tool involving 3180 6–7 year-olds attending 60 schools across the country. We demonstrated that transmission of *W*. *bancrofti* has been interrupted in all 21 districts.

**Conclusions:**

We conclude that LF transmission may have been interrupted in The Gambia through the extensive use of insecticidal nets for malaria control for decades. The growing evidence for the impact of malaria vector control activities on parasite transmission has been endorsed by WHO through a position statement in 2011 on integrated vector management to control malaria and LF.

## Author Summary

The prevalence of lymphatic filariasis (LF), in The Gambia was among the highest in Africa in the 1950s when about 50% of the adult population was positive for microfilaraemia. However, surveys conducted in 1975 and 1976 revealed a dramatic decline in LF endemicity in the absence of systematic treatment with anti-filaria medicines. This decline in LF prevalence in all villages was partly attributed to a significant drop in human-mosquito contact through a sustained reduction in rainfall in the 1960s and 1970s and the widespread use of insecticidal nets to protect against malaria. We asked the question in 2013 whether the rapid scale up in the use of insecticidal nets for malaria control in Gambia had resulted in the interruption of LF transmission. In this paper we present the results of three independently designed filariasis surveys conducted over a period of 17 years (1997–2013), and involving over 6000 subjects in 21 districts across all administrative divisions in the country. In 2001, tests performed to detect circulating filarial antigens (CFA) in serum samples collected between 1997 and 2000, in three divisions, failed to show positive individuals from two that were previously highly endemic for the LF. Results of the second survey conducted in 2003 indicated that five of the 21 districts were slightly endemic for LF with CFA rates of 1% or 2% but MDA was never implemented in The Gambia. The results of our final survey conducted in 2013 were unequivocal in confirming the absence of transmission of LF in all 21 districts surveyed using WHO recommended statistically robust and validated tool known as transmission assessment survey (TAS). The Gambia achieving a non-endemic status for LF represents a significant step in the efforts to shrink the filariasis endemicity map and demonstrates the value of cross sector approaches in disease control.

## Introduction

The Gambia is among 73 countries currently considered endemic for lymphatic filariasis (LF) by the World Health Organization [1]. LF, a neglected tropical disease (NTD), is a debilitating mosquito-borne nematode infection that affects 120 million people in low and middle income countries where 1.4 billion people are exposed to the parasites [1]. *Wuchereria bancrofti*, the causative agent of LF in The Gambia, is responsible for over 90% of the LF infections worldwide; *Brugia malayi* and *Brugia timori* account for the remaining infections and have a distribution restricted to the southeast Asian region [2]. The LF parasites are carried by various species of mosquito vectors from the genera *Anopheles*, *Aedes*, *Culex* and *Mansonia* but in sub-Saharan Africa, *Anopheles* species are the principal vectors[3]. There is no evidence that *Culex* species play any significant role in West Africa where the malaria vectors, *An*. *gambiae* s.l and *An*. *funestus*, are also the vectors of *W*. *bancrofti* [3–9]. In 1997, LF was targeted for elimination when the World Health Assembly adopted Resolution WHA 50.29 calling for the elimination of the disease as a public health problem globally [10]. In 2000, WHO, in collaboration with pharmaceutical companies and implementing partners launched the Global Programme to Eliminate LF (GPELF) as a disease specific intervention initiative to interrupt transmission and alleviate morbidity[11]. The GPELF has two strategic objectives for achieving this goal: 1) interruption of parasite transmission through mass drug administration (MDA) using albendazole in combination with either ivermectin or diethylcarbamazine citrate (DEC) and 2) morbidity management and disability prevention (MMDP) by providing access to care for those who suffer clinical manifestations of LF in endemic areas.

By 2012, 56 of the 73 countries where LF is considered endemic had started implementing MDA to eliminate the disease [1]. Among countries implementing MDA, only 13 countries had completed at least five rounds of MDA and moved to post-MDA surveillance phase [1]. Nevertheless, the scale up in the use of insecticidal nets and vector control in Africa, where 17 countries including The Gambia are yet to start MDA, has significantly reduced mosquito densities in many of these countries and contributed to a significant reduction in malaria prevalence [12, 13] and a possible decline in filariasis endemicity [3, 7, 14, 15]. In Solomon Islands where LF was transmitted by *Anopheles* mosquitoes, anti-mosquito measures to control malaria resulted in the interruption of LF transmission in the absence of MDA [16–18].

The Gambia had historically high prevalence of LF as described by Hawking [4, 5] in his historical reviews of the distribution of filariasis in Africa where he stated that the prevalence of *W*. *bancrofti* among adults in the Gambia in the 1950s [19, 20] was about 50%—one of the highest in the world. Prevalence surveys conducted in 17 villages across the country in 1975 and 1976 reported village specific microfilaraemia (MF) rates for people ≥15 years ranging from 2.9% to 26.9%; and the apparent decline after 25 years was mainly attributed to a reduction in mosquito density[21]. Nevertheless, MF positive children (<15 years) were present in many villages in the Upper River, Western and Lower River divisions where LF was still highly endemic.

WHO estimates that 1.2 million people require preventive chemotherapy against LF in The Gambia [22]. In line with the growing momentum to shrink the map of LF endemicity, we performed transmission assessment surveys (TAS) in The Gambia in May and June 2013 to determine if the widespread use of insecticidal bed nets and other vector control efforts over the past decades had eliminated transmission of *W*.*bancrofti* in the absence of MDA [12, 14]. The increasing momentum to take the “neglected” out of NTDs is driving public and private partners including drug companies, donors, and governments committed to what is now referred to as the 2012 London Declaration to shrink the NTD map and eliminate or eradicate by 2020 ten NTDs including LF [23]. The commitments made to date have been centred on ensuring the supply of drugs needed to implement preventive chemotherapy with limited emphasis on alternative intervention strategies such as vector control through the use of long lasting insecticidal nets (LLINs) or indoor residual spraying. Demonstrating the interruption of active transmission of *W*.*bancrofti* in The Gambia, in the absence of MDA, could have wide ranging public health and policy implications with regard to alternative strategies and synergies between malaria and NTD control programmes. In this paper we report the application of a new WHO methodology, the Transmission Assessment Survey (TAS), to validate the interruption of transmission in the absence of MDA against the disease in The Gambia after two mapping surveys carried out 15 years earlier suggested a dramatic decline in LF endemicity following a rapid decline in transmission and the possibility that elimination in the country could be achieved. The results presented here were collected over a period of 17 years (1997–2013), from three independently designed surveys, involving over 6000 subjects in 21 districts across all administrative divisions in the country.

## Materials and Methods

### Study area

**Fig 1:**
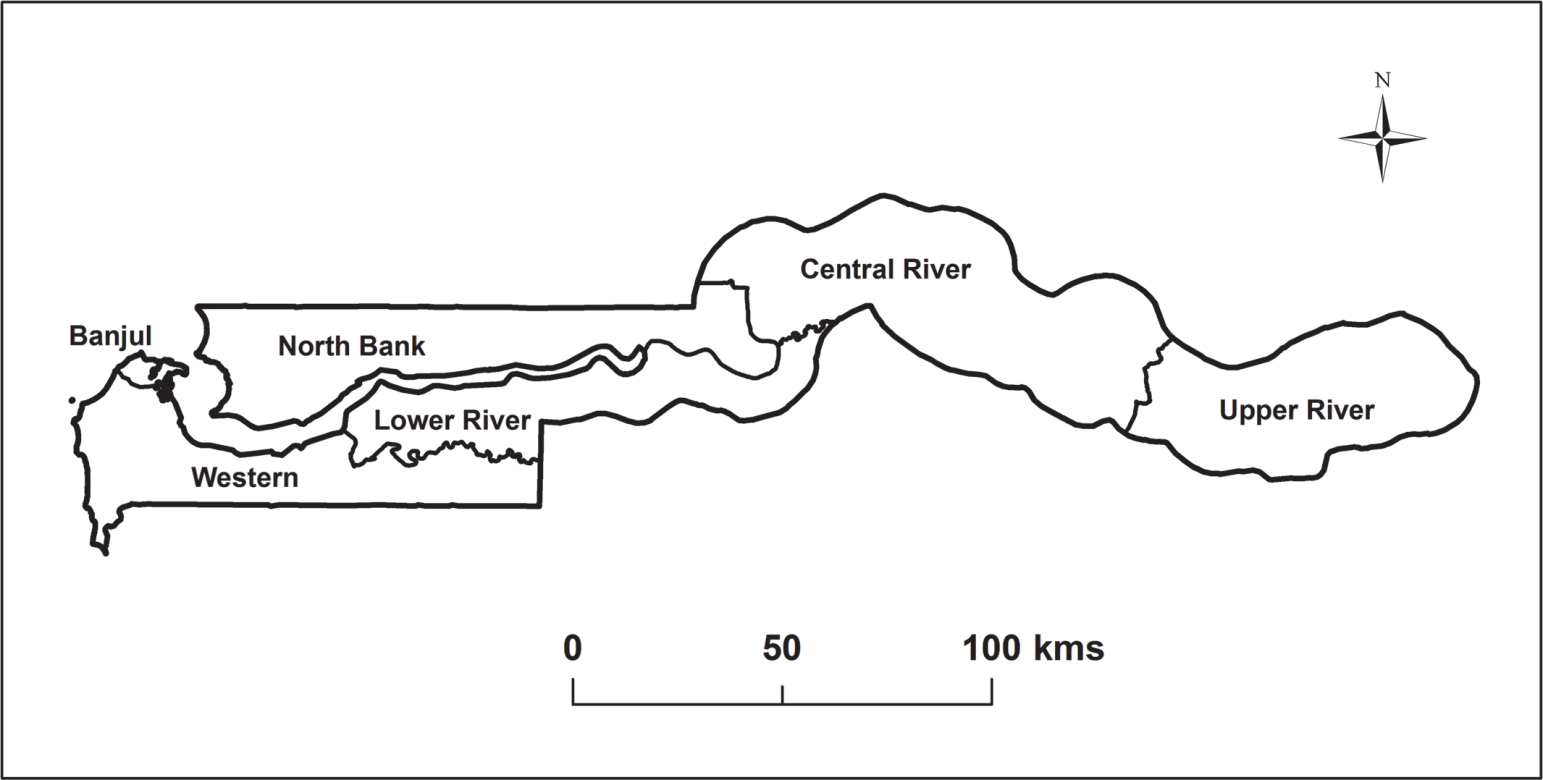
Map of The Gambia showing the historical boundaries for the six administrative divisions recognised before 2000.

The Gambia is a country in West Africa situated on either side of the Gambia River which flows through the country's centre and empties into the Atlantic Ocean. It lies between latitudes 13° and 14°N, and longitudes 13° and 17°W, and stretches inland for approximately 400 km. The Gambia is less than 48.2 km wide at its widest point, with a total area of 11,295 km^2^ and is surrounded by Senegal. It is the smallest country on mainland Africa with a population of 1,882,450. The climate is typically sub-Sahelian with one short rainy season from June to October. The country is presently divided into eight administrative divisions, including the national capital, Banjul. Prior to 2000, there were only 6 divisions in the country: Banjul, Western, North Bank, Lower River, Central River and Upper River. To retain geographic context with regard to the historical distribution of LF in The Gambia we will adhere, in this paper, with the former administrative boundaries demarcating 6 administrative divisions as shown in Fig. 1.

### Study design and blood sampling surveys

**Fig 2:**
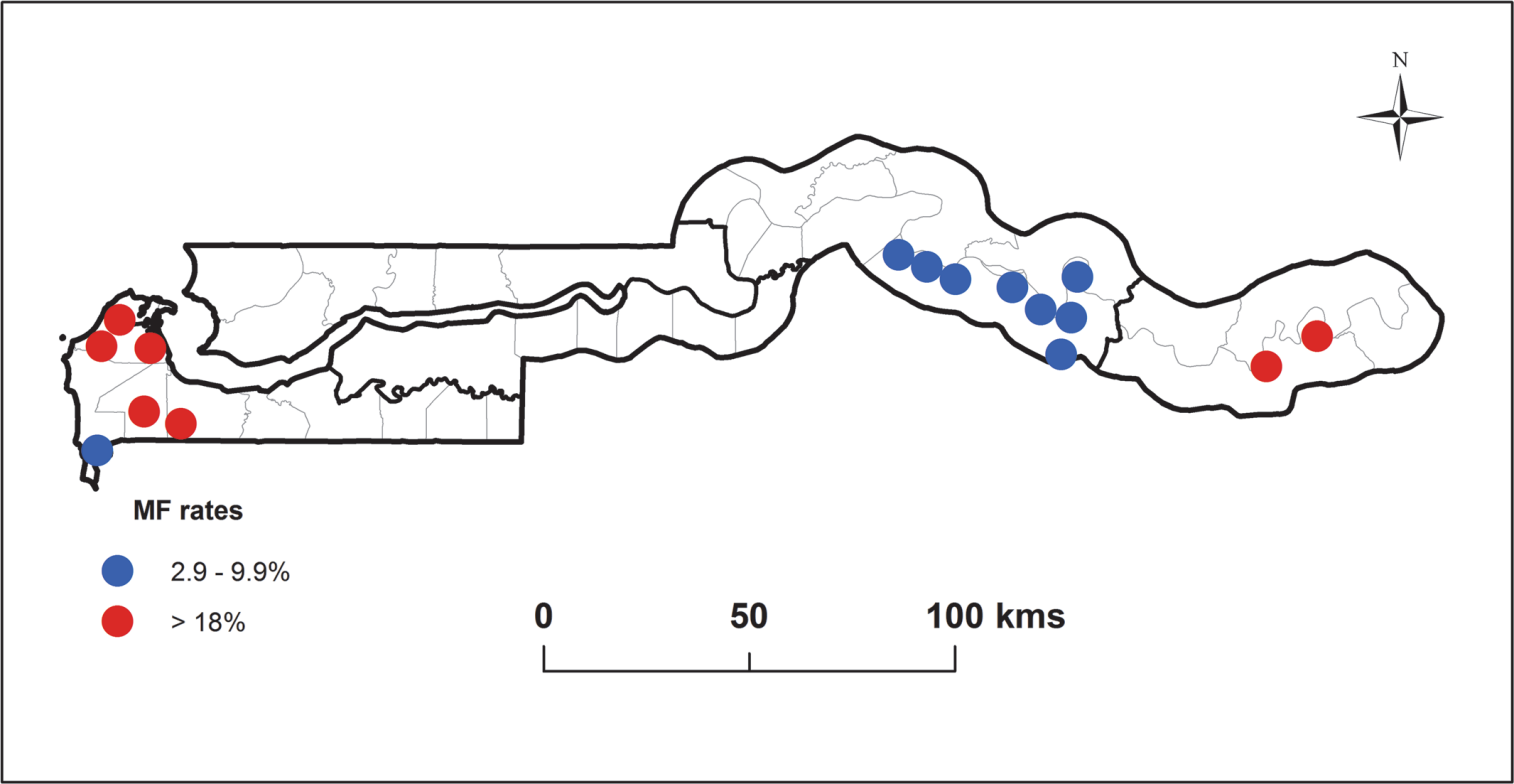
Map of The Gambia showing the location and microfilaria (MF rates in 15 villages surveyed in 1975 and 1976 (adapted from Reference #20) in areas of low transmission (Blue) where MF rates were less than 10% (Upper River and Western Divisions) and high transmission (Red) where MF rates where greater than 18% (Central River Division).

This is an observational study comparing historical prevalence from studies conducted in the 1950’s and 1970’s (Fig. 2) with 3 cross sectional surveys conducted in 2001, 2003 and 2013 examining LF infection through the distribution of circulating filarial antigen (CFA) positive individuals, involving over 6000 children and adults in 21 districts across all administrative divisions in The Gambia.

1. In 2001, stored serum samples from people ≥12 years residing in the previously highly endemic Divisions (North Bank, Upper River and West Coast) between 1997 and 2000 were tested for CFA. The serum/plasma samples were initially collected for malaria studies and stored in the British Medical Research Council (MRC) Laboratories in Fajara, The Gambia. The MRC laboratories established in The Gambia in 1947, carried out the first LF surveys in the country in 1951 but their primary focus for research is malaria. They routinely perform malaria surveys for several projects storing serum samples which can be processed for other parasites.
2. In 2003, a national LF mapping survey was carried out by the Ministry of Health and Social Welfare (MOHSW) in 30 high risk villages in all administrative divisions across the country to determine LF distribution and endemicity, and identify implementation units eligible for MDA. High risk communities were identified based on criteria described in the WHO manual for monitoring and evaluation (M&E) of LF programs [24], including historical data on LF, presence of diseased people and ecological suitability for mosquito breeding. MDA was, however, never implemented as the 2003 mapping results were not presented to the WHO Regional (African) Programme Review Group (RPRG) for review and determination of the need for MDA. This survey was based on sampling about 100 people in each high risk village purposely selected according to WHO mapping guidelines developed in 2000 [25].
3. In 2013, a school based TAS involving 60 randomly selected schools, was carried out as described below, to verify the absence of transmission after the remarkable decline towards extinction in CFA rates revealed by the 2003 survey results when compared with historic data presented in Fig. 2[21].

#### Transmission assessment surveys

A transmission assessment survey (TAS) protocol, designed for post MDA surveys, was used in this study to determine incidence of LF in the absence of MDA and to verify the absence of transmission after the remarkable decline towards extinction in CFA rates revealed during the 2003 survey. Molyneux and colleagues [26] discussed disease eradication, elimination and control and the need for accurate and consistent usage and defined elimination as the reduction of incidence to zero.

The design of the TAS described in the WHO M&E manual for national elimination programmes [24, 27], is based on lot quality assurance sampling (LQAS) with the sampling method used in the evaluation units (EU) being determined by the net primary school enrolment rate, the target population size and number of schools. Based on that, a random cluster sampling was chosen with schools as the sampling sites. A Survey Sample Builder (SSB) tool was used to determine sample size and sampling intervals. School surveys were conducted in all divisions as the net primary enrolment rates exceeded 75% (74%-78%). All children in grades 1 and 2 were eligible including a small proportion of those outside the 6–7 age range. Two evaluation units (EU) were created according to LF endemicity based on historical data as described above. The high transmission divisions of Western 1, North Bank, Lower River and Upper River were grouped into EU1 while Banjul, Western 2 and Central River Divisions were grouped into EU 2. Western 1 and Western 2 were previously part of the Western Division.

The sample sizes generated by the SSB were 30 schools clusters for each EU and 1552 children with a critical cut-off of 18 CFA positive children for the EU1 and 1556 children with a critical cut-off of 18 for EU2. Children who had not provided assent or parental custodian agreement, the severely sick and those who had lived in the community for less than two years were excluded from the TAS survey.

### Immunochromatographic tests (ICT) for CFA

The rapid Immunochromatographic tests (ICT) were used to test samples collected during the three surveys described above. At the point of care, the ICT card test was performed on whole blood or serum as described in the WHO monitoring and impact assessment manual [24] and following manufacturer’s instructions. The ICT cards used in 2001 and 2003 were produced by AMRAD ICT, New South Wales, Australia[28] while the cards used for the TAS surveys in 2013 were manufactured by Binax NOW Filariasis ICT card test (Alere Inc., Scarborough, ME). Both the AMRAD and Binax NOW ICTs were based on the same reagents and required the same quantify of sample volume (100 μl blood/serum) for testing. A positive [28]antigen control was used to test the validity of the ICT cards before the start of the survey in 2013.

### Data entry and analysis

Data generated before 2013 was managed through a simple spread sheet that calculated percentages for comparative analysis. The TAS data was managed through a Microsoft Access based data management system specifically developed by the Centre for Neglected Tropical Diseases (CNTD) in the Liverpool School of Tropical Medicine (LSTM) to support the cluster survey. Data entry was carried out by two independent clerks using a double entry system that automatically compared the two entries to detect and reconcile any discrepancies. The TAS critical cut-off value represents the threshold of infected individuals below which transmission is expected to be no longer sustainable, even in the absence of interventions.

If the total number of positive cases is at or below the critical cut-off, the EU ‘passes’ the survey and it is considered that transmission will no longer be sustainable. If the total number of positive cases is above the critical cut-off transmission is still ongoing in the evaluated unit[24]. TAS sample sizes and critical cut-off values are powered so that the EU has at least a 75% chance of passing if the true antigen prevalence is half the threshold level (2% for *Culex*, *Anopheles*, and *Mansonia* vector areas, and 1% for *Aedes* vector areas). In addition, there is no more than a 5% chance of passing if the true prevalence is greater than or equal to the threshold level.

### Ethical approval and consent procedures

Ethical clearance for the study was granted by The Medical Research Council (MRC) Laboratories Gambia Scientific Coordinating Committee, the LSTM Research Ethics Committee (Research Protocol (11.89R) and the Gambian Epidemiology and Disease Control Unit (EDC), of the MOH (correspondence 23 August 2012). After approval for TAS from the Education Ministries and school authorities, the MOHSW team met with the Head Master of each school to obtain permission to conduct the survey and to schedule a date for meeting with the parents. The survey team explained the purpose of the surveys and received oral consent from teachers and parents. Non-consenting parents or non-assenting children were not included in the survey. All individuals could drop out from the study any time during the study.

## Results

### Historical

The results of the surveys described here are best presented in the context of the historical distribution and endemicity of LF that informed the design of the surveys carried out to determine the pattern of LF burden and distribution in the absence of MDA. Fig. 2, adapted from the results of Knight [21] shows the burden and distribution of MF in 15 villages across five of the 6 divisions involved in this study carried out in the 1970s. Villages with MF rates of 18% or higher were found only in the Upper River and Western divisions. The MF rates in study villages in the other divisions were lower than 10%.

### Initial endemicity assessment: 1997–2000

**Fig 3:**
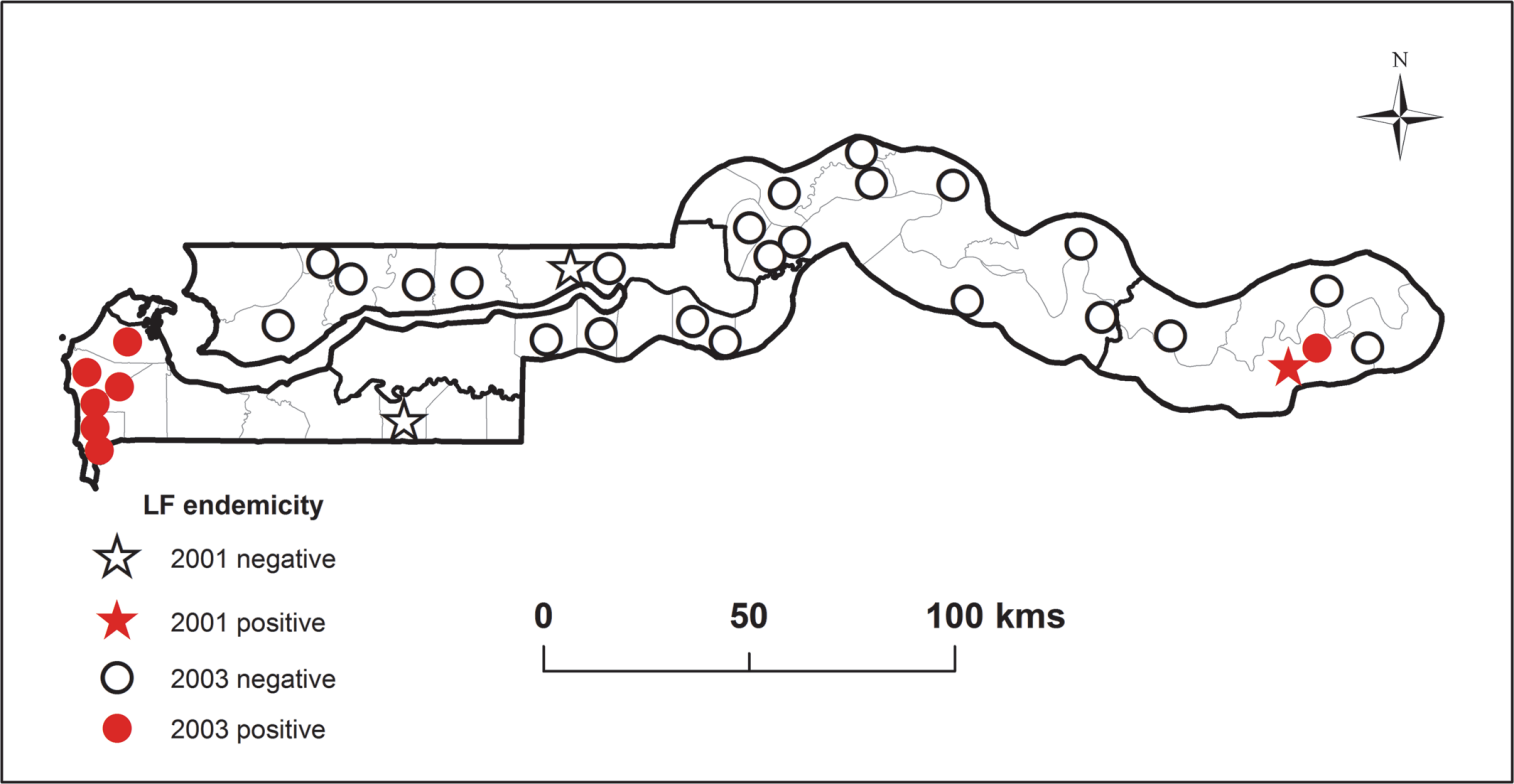
The lymphatic filariasis (LF) endemicity map for The Gambia showing locations of villages where at least one of the ~100 individuals tested during the mapping surveys was positive for CFA (Red dot or star) and villages where all ~100 tested individuals were found negative (White dot or star). The dots and stars represent villages surveyed during 2001 and 2003 respectively.

The initial assessment of LF endemicity was carried out in the Upper River and Western Divisions historically known to be highly endemic and the North bank where the MRC malaria project was based. In 2001 a total of 268 stored serum samples collected between 1997 and 2000 from people ≥12 years residing in three villages (Basse, Brefet, Farafenni) in the three divisions, were tested with ICT for the presence of CFA. CFA positive individuals (n = 8) were only found in Basse village in the Upper River Division where 100 samples were tested. All 68 samples from Brefet (Western Division) and 100 from Farafenni (North Bank Division were negative for CFA. Results are shown as stars in Fig. 3. The red star indicates the location of the village where CFA positives were found in the Upper River Division.

The 8% CFA rate observed for the endemic division corresponds to between 2% and 4% MF rate [29] indicating 89–91% reduction in MF rate over 20 years in the absence of MDA in comparison to the 21% (32/148) MF rates observed in the same area in 1976 by Knight [21]. The MF rates in the Western division decreased from 15% (66/439) in 1976 to 0% (0/68) in 2000.

### Nationwide mapping survey: 2003

The results of the national filariasis mapping survey conducted in 2003 are presented in Fig. 3 with circles showing the location and endemicity status of the 30 villages tested in 21 Districts across all 6 divisions. Altogether, 3113 individuals age ≥15 years were tested from the different divisions: Central River (1156), Lower River (391), North Bank (682), Upper River (423) and Western (561). CFA positive individuals were found only in 9 villages located in 5 districts outside the Central River Division where historically the MF rates had been low (Fig. 2). Among the 9 villages with CFA positives, 6 were located in the Western and Upper River Divisions where the highest MF rates were found during the 1975 and 1976 surveys (Figs 2 and 3). The remaining three villages with CFA positives were located in the North River and Lower River divisions not included in the historical surveys.

### Transmission assessment surveys: 2013

**Fig 4:**
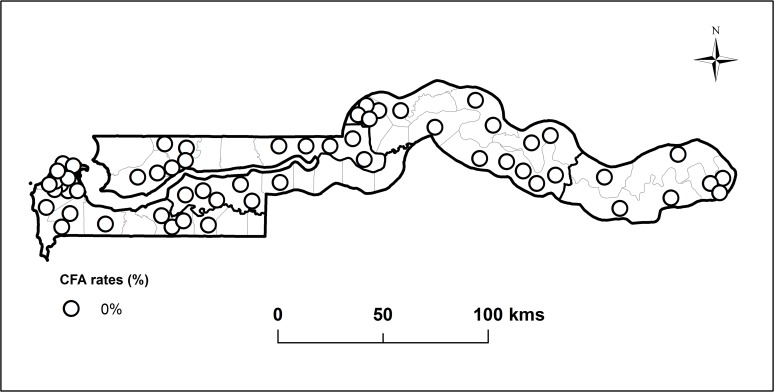
Map of The Gambia showing the location of 60 schools surveyed during the Transmission Assessment Surveys (TAS) carried out in 2013 and revealing no circulating filarial antigen (CFA) positive individuals in all schools indicated by the white dots.

In 2013, TAS was conducted in 60 randomly selected schools in all Divisions in The Gambia, 30 in each of the two EUs, to assess if active transmission of *W*.*bancrofti* was ongoing. In total, 3180 6–7 year-old children (1509 boys and 1671 girls) were tested using ICT and none was found to be CFA positive. 1516 children were tested in EU1 and 1664 on EU2. Fig. 4 shows the location of schools tested during the TAS.

The changing pattern of LF infection rates determined by night blood and ICT surveys across the 6 administrative divisions in the Gambia between 1951 and 2013 is presented in Table 1.

**Table 1:** The changing pattern of LF infection rates determined by night blood and ICT surveys across the 6 administrative divisions in The Gambia between 1951 and 2013.

Year	Administrative Division	Age group examined	Survey type and number of sampling sites	Site specific infection rate	Notes	References #
1951–1954	Western	All age groups	Night blood surveys in 4 villages	36–50%	1951 survey in area hyperendemic for malaria. No infections found in children younger than 4.5 years. 1954 survey also failed to detect mf in children <4 years.	4, 19–21,30–31
1974–1976	Western, Central, and Upper River	≥3 years	Night blood surveys in 15 villages	1.5% (≥ 3 yrs), 6.7–22.7% (≥ 5 yrs), 2.9–26.9% (≥ 15 yrs)	Night blood surveys	21
1997–2000	North Bank, Upper River and Western	≥12 years	ICT surveys on samples from 3 villages	0–8%	ICT tests performed on stored serum samples collected for a malaria project.	Current study
2003	Banjul, Central, Lower River, North Bank, Upper River and Western	≥15 years	ICT mapping surveys in 30 villages	0–3%	Mapping survey carried out in collaboration with the Ministry of Health and Social Welfare, Gambia.	Current study
2013	Banjul, Central, Lower River, North Bank, Upper River and Western	6–7 years	ICT TAS surveys in 60 schools	0%	Transmission assessment survey conducted in collaboration with the Ministry of Health and Social Welfare, Gambia	Current study

## Discussion

The TAS performed in this study verified the absence of transmission in all administrative divisions that were previously highly endemic for LF. The prevalence of *W*.*bancrofti* in The Gambia was among the highest in Africa, based on historical data [20, 21, 30–32] and reported in the first global atlas of LF compiled by Michael and Bundy in 1997 [33] and the African LF risk map of Lindsay and Thomas [30]. Village specific MF rates reported in studies carried out in the 1950s, among people 10 years or older, varied between 24.1% and 48.4% [20, 31, 32] and Hawking[4, 5] later reported that the prevalence of LF among adults during the early 1950s was about 50%. Based on our current knowledge of the relationship between MF rates and antigen prevalence such figures suggest that the majority of adults were infected with the parasite in the 1950’s. Night blood surveys conducted in 15 villages in 1975 and 1976 revealed MF rates between 2.9% and 26.9% among adults ≥15 years [21]. The corresponding high prevalence of LF morbidity observed in children and adults in The Gambia in the 1970s confirmed that transmission had continued in the twenty years since the first surveys in the 1950s [21]. In adults older than 15 years, morbidity presented in the form of adenolymphangitis (13.8%–20.0%), lymphoedema (5.3%–10.0%) and hydrocele (11.7%–35.3%) [21]. Late filarial disease in the form of elephantiasis and hydrocoele was also observed in children younger than 15 years [21] suggesting early exposure and high levels of transmission.

A multi country operational research study undertaken in 11 EUs in Africa and Asia in 2011 and 2012, to test the value and practicality of the TAS tool, concluded that it was a practical and effective tool for evaluating interruption of transmission [27]. In 10 of 11 EUs surveyed during the study, the number of CFA positive cases was below the critical cut-offs of 18, leading to a cessation of MDA in these communities.

The low CFA rates (≤3%) observed in the previously highly endemic divisions, during the 2003 mapping surveys, therefore implies a significant reduction in MF rates in patent infection in The Gambia between 1976 and 2003. A 9% CFA rate observed by Gass and colleagues [29] in a highly endemic area in Ghana corresponded to a 2% MF rate. Also, previous studies have shown that stored sera can remain antigen positive even after 10 years in storage [34]. In 2013, all 3180 children tested during the TAS surveys were found to be negative for CFA suggesting an interruption of the transmission of *W*.*bancrofti* in the absence of MDA. Results of the mapping surveys conducted in 2003 indicated that MDA for LF was required for 5 districts where CFA rates were 1% or higher [24]. No ‘endemic’ districts were found in the Central Division where the intensity of transmission was historically lower [17]. MDA was however not implemented by the MOHSW [1].

LF is a chronic infection perpetuated by the continuous production of microfilaria by lymphatic dwelling adult worms that can live for 4–6 years [35]. If competent mosquito vectors are present in adequate numbers, transmission will continue for as long as the MF density is maintained above a certain threshold. The host-parasite relationship in *W*.*bancrofti* transmission dynamics has been subjected to analysis using mathematical models and two infection patterns, limitation and facilitation, have emerged depending on the vector species involved [36–39]. Limitation, due to culicine transmitted filariasis, and facilitation, due to anopheline vectors have been discussed in detail [3, 40–43]. The models predict that in culicine transmitted-LF (limitation) the number of ingested microfilariae is always greater than the constant predicted by a straight line proportionality relationship so that even at low MF densities parasite uptake by mosquitoes occurs. Conversely, with anopheline-transmitted filariasis (facilitation), as in The Gambia, a lower critical threshold exists below which the number of ingested microfilariae decreases to the constant ratio. Under these conditions, transmission is not sustained and the infection will be eliminated. The critical threshold below which *Anopheles*-transmitted LF will be eliminated can be achieved either by reducing the density of microfilaria or the density of mosquitoes [44]. In The Gambia, where onchocerciasis is non endemic [45], there was never a systematic community-wide filariasis treatment that would have resulted in the reduction of *W*. *bancrofti* MF density [21]. The most likely explanation for our observation that transmission has been interrupted is the reduction of vector density through the widespread use of insecticide treated bednets that scaled up dramatically after 2003 and became more effective with the introduction of LLINs [12, 46] for malaria control. A Sahelian drought in neighbouring countries resulted in a significant reduction in rainfall in the Gambia that probably affected mosquito numbers and LF transmission in the late 1960s and 1970s [6, 21]. Nevertheless LF was still highly endemic in the Western and Upper River divisions during the late 1970s [6, 21].

The vectors of malaria in The Gambia are also involved in the transmission of LF with Anopheles arabiensis, *An*. *gambiae s*.*s*., *An*. *melas* and *An*. *funestus* acting as the principal carriers of *W*. *bancrofti*. *Culex* mosquitoes probably play no role in the transmission of LF in The Gambia as is the case in other countries in West Africa where only *Anopheles* mosquitoes act as vectors of *W*. *bancrofti*. Despite the early elucidation of the biology and behaviour of *Anopheles* mosquitoes in The Gambia, there were no systematic vector control activities in the country until insecticide treated bednets were introduced in the 1990s [21]. The report of the Liverpool School of Tropical Medicine expedition to The Gambia in 1901 recommended measures to prevent mosquito breeding in the capital, Bathurst [47]. In 1904, an ordinance covering the prevention of mosquito breeding and establishing posts for a sanitary inspector and a team of labourers to implement vector control was introduced. However, outside Bathurst no systematic attempts were made to control mosquitoes until insecticide treated bednets were introduced in the 1990s and scaled up gradually [47]. The main vectors of LF in the Gambia, are highly susceptible to insecticidal nets and indoor residual spray (IRS). The use of IRS to control malaria in the 1970s and 1980s interrupted LF transmission in Solomon Islands, parts of Papua New Guinea, Indonesia and Togo [3]. Community-wide use of long lasting insecticidal nets recently interrupted the transmission of LF in parts of Nigeria[48] and Papua New Guinea [49]. Improvements in living standards and housing characteristics probably contributed to the elimination of LF in Seychelles, Cape Verde, and Mauritius where *Culex* mosquitoes were the main vectors [50]. There is however little evidence that improvement in living standards have significantly impacted LF transmission in rural areas in Sierra Leone where LF was endemic in all districts before MDA commenced in 1998 [51].

Knight [21] who performed MF surveys in 1975 and 1976 attributed the decline in MF rates between 1950 and 1976 to a reduction in human-mosquito contact rates through decreased rainfall, improved standard of living and vector control against malaria and nuisance mosquitoes through bednets,at that time not impregnated with insecticide. Vector control in the form of IRS with DDT conducted during the malaria eradication campaign in Solomon Islands from 1974 to 1977 decreased the MF prevalence from 22% to zero without the use of anti-filarial drugs over a period of four years [17, 18].

As a result of Global Fund and PMI funding for bednet implementation, a rapid scale up in ITN coverage has occurred in many countries in Africa since 2003 when only Eritrea, Malawi, The Gambia, and Sao Tome and Principe had ITN ownership coverage greater than 20%[12]. Analysing data from suppliers of ITN to countries, National Malaria Control Program (NMCP) reports of ITNs distributed to health facilities and implementation partners and household survey data, Flaxman and co-workers[12] calculated insecticidal net ownership coverage for 44 countries in Africa between 2003 and 2008. By the middle of 2008, 8 countries including The Gambia had ITN ownership coverage of 60% or greater. There were substantial increases from 2003 in the delivery of interventions against malaria, including distribution of insecticidal nets, in The Gambia [52]. Investigating the changes in malaria indices in the country, and the causes and public-health significance of these changes, Ceesay and collaborators [13] reported that the proportions of malaria-positive slides decreased by 82% and concluded that a large proportion of the malaria burden has been alleviated in The Gambia. This remarkable reduction in malaria parasite positive individuals would confirm our views given that malaria and *W*.*bancrofti* have common *Anopheles* vectors, the absence of any detectable *W*.*bancrofti* in our sample of over 3000 children throughout the country is similarly due to the widespread use of insecticide impregnated nets and now LLINs.

Previous trials of chemoprophylaxis and insecticidal nets against malaria in The Gambia have shown reductions in all-cause morbidity and mortality greater than that directly attributable to malaria [13]. Improved treatment against malaria contributed to the reduction in malaria transmission but according to Ceesay and colleagues [13] the most significant factor was the increase of coverage of insecticidal nets because of support from the Global Fund—awarded in 2003 and implemented from 2004 onwards—for free distribution of ITNs to pregnant women and mothers of children younger than 5 years, which according to Ceesay and co-workers accounted for 55% of the population. A nationwide survey carried out to investigate the use of ITNs in rural areas of the Gambia in 1991, showed that 58% of beds had a net [46]. Bednet usage was higher in the Central Region (76%), than in the Western and Eastern Regions (both 51%)[46]. This could partly explain the difference in LF endemicity between the Central and Western divisions observed during this study.

In Kenya, one of the few African countries where the coverage of insecticidal nets exceeded 40% in 2007[12], LF prevalence continued to decrease even when MDA was interrupted for two years [53]. In Sierra Leone, where bednet usage was traditionally low [54] and scaling up of insecticide-treated nets was among the slowest in Africa [12], LF remains endemic in many parts of the country after 10 years of MDA with ivermectin for onchocerciasis and more recently for LF [45, 55]. Onchocerciasis is not endemic in The Gambia and hence mass treatment with the ivermectin has never been implemented [45]. Comprehensive reviews on the advances in knowledge of vector ecology, vector-parasite relationships, and both empirical and theoretical evidence regarding vector management to determine the role of vector control in the GPELF have concluded that MDA activities can be synergised with vector control and LF elimination will be easier to achieve [3, 7, 14, 15, 56]. Recent studies on the impact of insecticide treated nets on LF transmission in PNG [49] and Nigeria[48, 57] showed that using insecticidal nets alone in the absence of MDA interrupted the transmission of LF in highly endemic areas with MF rates over 50%.

Failure to find evidence of transmission to children of *W*. *bancrofti* in The Gambia in 2013 over a 6–7 year period suggests that transmission has been interrupted and the most likely cause was the extensive use of insecticide treated nets for malaria control over the last two decades built upon the causes of the decline observed [17] between the 1950 and 1970s. The growing evidence for the impact of malaria vector control activities on LF transmission was endorsed by WHO through a position statement in 2011 on integrated vector management (IVM) to control malaria and lymphatic filariasis [58]. IVM is promoted by WHO to strengthen partnerships and cross sector approaches to the control of mosquito-borne diseases like malaria and LF [15, 58]. In 2014, the Nigerian government converted these evidence based approaches into policy by launching a coordinated plan to eliminate malaria and lymphatic filariasis through the use of long lasting insecticidal nets (http://www.afro.who.int/en/nigeria/press-materials/item/6286-nigeria-launches-the-malaria-and-lymphatic-filariasis-co-implementation-guidelines.html). MDA may not be required to achieve elimination of LF in The Gambia but surveillance processes prescribed for countries during the post MDA phase, including morbidity management and disability prevention will be necessary, to acquire a non-endemic status that demands verification. The Gambia achieving non-endemic status for LF will represents huge progress in the global efforts to shrink the filariasis endemicity map and demonstrates the value of cross sector approaches in disease control. One more country will be removed from the list of LF endemic countries and 1.2 million people will be declared to be no longer at risk for the disease

## Supporting Information

S1 Checklist STROBE Checklist. (DOC)

S1 Text Additional survey results. Survey results for all schools that participated in the transmission assessment surveys.
(PDF)

## References

[1] WHO. Global programme to eliminate lymphatic filariasis: progress report for 2012. Releve epidemiologique hebdomadaire / Section d'hygiene du Secretariat de la Societe des Nations = Weekly epidemiological record / Health Section of the Secretariat of the League of Nations. 2013;88(37):389–99. 24073461

[2] Taylor MJ, Hoerauf A, Bockarie M. Lymphatic filariasis and onchocerciasis. Lancet. 2010;376(9747):1175–85. 10.1016/S0140-6736(10)60586-7 20739055

[3] Bockarie MJ, Pedersen EM, White GB, Michael E. Role of vector control in the global program to eliminate lymphatic filariasis. Annual review of entomology. 2009;54:469–87. 10.1146/annurev.ento.54.110807.090626 18798707

[4] Hawking F. The distribution of Bancroftian filariasis in Africa. Bulletin of the World Health Organization. 1957;16(3):581–92. 13472412 PMC2538322

[5] Hawking F. The distribution of human filariasis throughout the world. Part III. Africa. Tropical diseases bulletin. 1977;74(8):649–79. 919037

[6] Bryan JH, Southgate BA. Factors affecting transmission of Wuchereria bancrofti by anopheline mosquitoes. 1. Uptake of microfilariae. Transactions of the Royal Society of Tropical Medicine and Hygiene. 1988;82(1):128–37. 3051542 10.1016/0035-9203(88)90286-6

[7] de Souza DK, Koudou B, Kelly-Hope LA, Wilson MD, Bockarie MJ, Boakye DA. Diversity and transmission competence in lymphatic filariasis vectors in West Africa, and the implications for accelerated elimination of Anopheles-transmitted filariasis. Parasites & vectors. 2012;5:259. 23151383 10.1186/1756-3305-5-259 PMC3533928

[8] Brengues J. [Culex pipiens fatigans Wiedemann in tropical Africa: its importance and its control (author's transl)]. Medecine tropicale: revue du Corps de sante colonial. 1978;38(6):691–4. 370500

[9] Brengues J. La filariose de bancroft en Afrique de l’Ouest. ORSTOM No 79. 1975.

[10] Ottesen EA, Duke BO, Karam M, Behbehani K. Strategies and tools for the control/elimination of lymphatic filariasis. Bulletin of the World Health Organization. 1997;75(6):491–503. 9509621 PMC2487030

[11] Ottesen EA. The global programme to eliminate lymphatic filariasis. Tropical medicine & international health: TM & IH. 2000;5(9):591–4. 11044272 10.1046/j.1365-3156.2000.00620.x

[12] Flaxman AD, Fullman N, Otten MW Jr., Menon M, Cibulskis RE, Ng M, et al Rapid scaling up of insecticide-treated bed net coverage in Africa and its relationship with development assistance for health: a systematic synthesis of supply, distribution, and household survey data. PLoS medicine. 2010;7(8):e1000328 10.1371/journal.pmed.1000328 20808957 PMC2923089

[13] Ceesay SJ, Casals-Pascual C, Erskine J, Anya SE, Duah NO, Fulford AJ, et al Changes in malaria indices between 1999 and 2007 in The Gambia: a retrospective analysis. Lancet. 2008;372(9649):1545–54. 10.1016/S0140-6736(08)61654-2 18984187 PMC2607025

[14] Kelly-Hope LA, Molyneux DH, Bockarie MJ. Can malaria vector control accelerate the interruption of lymphatic filariasis transmission in Africa; capturing a window of opportunity? Parasites & vectors. 2013;6:39. 23433078 10.1186/1756-3305-6-39 PMC3599698

[15] van den Berg H, Kelly-Hope LA, Lindsay SW. Malaria and lymphatic filariasis: the case for integrated vector management. The Lancet infectious diseases. 2013;13(1):89–94. 10.1016/S1473-3099(12)70148-2 23084831

[16] Webber RH. Vector control of filariasis in the Solomon Islands. The Southeast Asian journal of tropical medicine and public health. 1975;6(3):430–4. 3855

[17] Webber RH. The natural decline of Wuchereria bancrofti infection in a vector control situation in the Solomon Islands. Transactions of the Royal Society of Tropical Medicine and Hygiene. 1977;71(5):396–400. 595094 10.1016/0035-9203(77)90037-2

[18] Webber RH. Eradication of Wuchereria bancrofti infection through vector control. Transactions of the Royal Society of Tropical Medicine and Hygiene. 1979;73(6):722–4. 395730 10.1016/0035-9203(79)90031-2

[19] Mc GI, Smith DA. A health, nutrition and parasitological survey in a rural village (Keneba) in west Kiang, Gambia. Transactions of the Royal Society of Tropical Medicine and Hygiene. 1952;46(4):403–27. 14958820 10.1016/0035-9203(52)90058-8

[20] McGregor IA, Gilles HM. Diethylcarbamazine control of bancroftian filariasis; follow-up of a field trial in West Africa. British medical journal. 1956;1(4962):331–2. 13284317 10.1136/bmj.1.4962.331 PMC1978876

[21] Knight R. Current status of filarial infections in The Gambia. Annals of tropical medicine and parasitology. 1980;74(1):63–8. 6990885 10.1080/00034983.1980.11687311

[22] WHO. Global Programme to Eliminate Lymphatic Filariasis: progress report 2000–2009 and strategic plan 2010–2020 World Health Organization (WHO/HTM/NTD/PCT/20106). 2010.

[23] (Editorial) L. Neglected tropical diseases: becoming less neglected. Lancet. 2014;383(9925):1269 10.1016/S0140-6736(14)60629-2 24725560

[24] WHO. Monitoring and epidemiological assesment of mass drug administration: a manual for national elimination programmes World Health Organization (WHO/HTM/NTD/PCT/20114). 2011.

[25] WHO. Operational Guidelines for Rapid Mapping of Bancroftian Filariasis in Africa. WHO/CDS/CPE/CEE/20009. 2000.

[26] Molyneux DH, Hopkins DR, Zagaria N. Disease eradication, elimination and control: the need for accurate and consistent usage. Trends in parasitology. 2004;20(8):347–51. 15246314 10.1016/j.pt.2004.06.004

[27] Chu BK, Deming M, Biritwum NK, Bougma WR, Dorkenoo AM, El-Setouhy M, et al Transmission assessment surveys (TAS) to define endpoints for lymphatic filariasis mass drug administration: a multicenter evaluation. PLoS neglected tropical diseases. 2013;7(12):e2584 10.1371/journal.pntd.0002584 24340120 PMC3855047

[28] Schuetz A, Addiss DG, Eberhard ML, Lammie PJ. Evaluation of the whole blood filariasis ICT test for short-term monitoring after antifilarial treatment. The American journal of tropical medicine and hygiene. 2000;62(4):502–3. 11220767 10.4269/ajtmh.2000.62.502

[29] Gass K, Beau de Rochars MV, Boakye D, Bradley M, Fischer PU, Gyapong J, et al A multicenter evaluation of diagnostic tools to define endpoints for programs to eliminate bancroftian filariasis. PLoS neglected tropical diseases. 2012;6(1):e1479 10.1371/journal.pntd.0001479 22272369 PMC3260316

[30] Lindsay SW, Thomas CJ. Mapping and estimating the population at risk from lymphatic filariasis in Africa. Transactions of the Royal Society of Tropical Medicine and Hygiene. 2000;94(1):37–45. 10748895 10.1016/s0035-9203(00)90431-0

[31] McGregor IA, Hawking F, Smith DA. The control of filariasis with hetrazan; a field trial in a rural village (Keneba) in the Gambia. British medical journal. 1952;2(4790):908–11. 12978380 10.1136/bmj.2.4790.908 PMC2021811

[32] McFadzean JA. Filariasis in Gambla and Casamance, West Africa. Transactions of the Royal Society of Tropical Medicine and Hygiene. 1954;48(3):267–73. 13169245 10.1016/0035-9203(54)90075-9

[33] Michael E, Bundy DA. Global mapping of lymphatic filariasis. Parasitology today. 1997;13(12):472–6. 15275135 10.1016/s0169-4758(97)01151-4

[34] Tisch DJ, Bockarie MJ, Dimber Z, Kiniboro B, Tarongka N, Hazlett FE, et al Mass drug administration trial to eliminate lymphatic filariasis in Papua New Guinea: changes in microfilaremia, filarial antigen, and Bm14 antibody after cessation. The American journal of tropical medicine and hygiene. 2008;78(2):289–93. 18256431 PMC2590750

[35] WHO. Accelerated work to overcome the global impact of neglected tropical diseases: A roapmap for implementation World Health Organization (WHO/HTM/NTD/20121). 2012.

[36] Bain O. Transmission des filarioses. Limitation des passages des microfilaires ingérées vers l'hémocèle du vecteur; interprétation. Annales de parasitologie humaine et comparée. 1971;46(613–631). 5170431

[37] Bain O, Brengues J. Transmission de la Wuchéreriose et de la sétariose bovine: étude histologique de la traversee de la paroi stomacale d'*Anopheles gambiae* A et *d'Aedes aegypti* par les microfilaires. Annales de parasitologie humaine et comparée. 1972;47:399–412. 4565488

[38] Pichon G. Relations mathématiques entre le nombre des microfilaires ingérées et le nombre des parasites chez different vecteurs naturels ou expérimentaux de filarioses. Cahiers ORSTOM, Série Entomologie médicale et Parasitologie. 1974;12:199–216.

[39] Pichon G. Limitation and facilitation in the vectors and other aspects of the dynamics of filarial transmission: the need for vector control against Anopheles-transmitted filariasis. Annals of tropical medicine and parasitology. 2002;96 Suppl 2:S143–52. 12625927 10.1179/000349802125002509

[40] Gambhir M, Bockarie M, Tisch D, Kazura J, Remais J, Spear R, et al Geographic and ecologic heterogeneity in elimination thresholds for the major vector-borne helminthic disease, lymphatic filariasis. BMC biology. 2010;8:22 10.1186/1741-7007-8-22 20236528 PMC2848205

[41] Snow LC, Bockarie MJ, Michael E. Transmission dynamics of lymphatic filariasis: vector-specific density dependence in the development of Wuchereria bancrofti infective larvae in mosquitoes. Medical and veterinary entomology. 2006;20(3):261–72. 17044876 10.1111/j.1365-2915.2006.00629.x

[42] Southgate BA, Bryan JH. Factors affecting transmission of Wuchereria bancrofti by anopheline mosquitoes. 4. Facilitation, limitation, proportionality and their epidemiological significance. Transactions of the Royal Society of Tropical Medicine and Hygiene. 1992;86(5):523–30. 1475823 10.1016/0035-9203(92)90096-u

[43] Wada Y, Kimura E, Takagi M, Tsuda Y. Facilitation in Anopheles and spontaneous disappearance of filariasis: has the concept been verified with sufficient evidence? Tropical medicine and parasitology: official organ of Deutsche Tropenmedizinische Gesellschaft and of Deutsche Gesellschaft fur Technische Zusammenarbeit. 1995;46(1):27–30. 7631124

[44] Webber RH. Can anopheline-transmitted filariasis be eradicated? The Journal of tropical medicine and hygiene. 1991;94(4):241–4. 1880825

[45] Amazigo U. The African Programme for Onchocerciasis Control (APOC). Annals of tropical medicine and parasitology. 2008;102 Suppl 1:19–22. 10.1179/136485908X337436 18718149

[46] D'Alessandro U, Aikins MK, Langerock P, Bennett S, Greenwood BM. Nationwide survey of bednet use in rural Gambia. Bulletin of the World Health Organization. 1994;72(3):391–4. 8062396 PMC2486695

[47] Greenwood BM, Pickering H. A malaria control trial using insecticide-treated bed nets and targeted chemoprophylaxis in a rural area of The Gambia, west Africa. 1. A review of the epidemiology and control of malaria in The Gambia, west Africa. Transactions of the Royal Society of Tropical Medicine and Hygiene. 1993;87 Suppl 2:3–11. 8212107 10.1016/0035-9203(93)90169-q

[48] Richards FO, Emukah E, Graves PM, Nkwocha O, Nwankwo L, Rakers L, et al Community-wide distribution of long-lasting insecticidal nets can halt transmission of lymphatic filariasis in southeastern Nigeria. The American journal of tropical medicine and hygiene. 2013;89(3):578–87. 10.4269/ajtmh.12-0775 23939708 PMC3771303

[49] Reimer LJ, Thomsen EK, Tisch DJ, Henry-Halldin CN, Zimmerman PA, Baea ME, et al Insecticidal bed nets and filariasis transmission in Papua New Guinea. The New England journal of medicine. 2013;369(8):745–53. 10.1056/NEJMoa1207594 23964936 PMC3835352

[50] WHO. Global Programme to eliminate lymphatic filariasis: progress report on mass drug administration, 2010. Releve epidemiologique hebdomadaire / Section d'hygiene du Secretariat de la Societe des Nations = Weekly epidemiological record / Health Section of the Secretariat of the League of Nations. 2011;86(35):377–88. 21887884

[51] Koroma JB, Bangura MM, Hodges MH, Bah MS, Zhang Y, Bockarie MJ. Lymphatic filariasis mapping by immunochromatographic test cards and baseline microfilaria survey prior to mass drug administration in Sierra Leone. Parasites & vectors. 2012;5:10. 22236419 10.1186/1756-3305-5-10 PMC3268710

[52] Noor AM, Mutheu JJ, Tatem AJ, Hay SI, Snow RW. Insecticide-treated net coverage in Africa: mapping progress in 2000–07. Lancet. 2009;373(9657):58–67. 10.1016/S0140-6736(08)61596-2 19019422 PMC2652031

[53] Njenga SM, Mwandawiro CS, Wamae CN, Mukoko DA, Omar AA, Shimada M, et al Sustained reduction in prevalence of lymphatic filariasis infection in spite of missed rounds of mass drug administration in an area under mosquito nets for malaria control. Parasites & vectors. 2011;4:90. 21612649 10.1186/1756-3305-4-90 PMC3125382

[54] Barnish G, Maude GH, Bockarie MJ, Eggelte TA, Greenwood BM. The epidemiology of malaria in southern Sierra Leone. Parassitologia. 1993;35 Suppl:1–4. 8233597

[55] Koroma JB, Sesay S, Sonnie M, Hodges MH, Sahr F, Zhang Y, et al Impact of three rounds of mass drug administration on lymphatic filariasis in areas previously treated for onchocerciasis in Sierra Leone. PLoS neglected tropical diseases. 2013;7(6):e2273 10.1371/journal.pntd.0002273 23785535 PMC3681681

[56] Molyneux DH, Hotez PJ, Fenwick A, Newman RD, Greenwood B, Sachs J. Neglected tropical diseases and the Global Fund. Lancet. 2009;373(9660):296–7. 10.1016/S0140-6736(09)60089-1 19167564

[57] Eigege A, Kal A, Miri E, Sallau A, Umaru J, Mafuyai H, et al Long-lasting insecticidal nets are synergistic with mass drug administration for interruption of lymphatic filariasis transmission in Nigeria. PLoS neglected tropical diseases. 2013;7(10):e2508 10.1371/journal.pntd.0002508 24205421 PMC3814337

[58] WHO. WHO position statement on integrated vector management to control malaria and lymphatic filariasis. Releve epidemiologique hebdomadaire / Section d'hygiene du Secretariat de la Societe des Nations = Weekly epidemiological record / Health Section of the Secretariat of the League of Nations. 2011;86(13):121–7. 21438441

