# Supplementary material for: Elimination of Lymphatic Filariasis in The Gambia
Source: PLoS Negl Trop Dis. 2015 Mar 18;9(3):e0003642. doi: 10.1371/journal.pntd.0003642 (PMC4364952; doi:10.1371/journal.pntd.0003642)
Supplement: S1 Text — Survey results for all schools that participated in the transmission assessment surveys. (PDF) [file pntd.0003642.s002.pdf]

NO	SCHOOL	LOCATION	DISTRICT	REGION	MALES	FEMALES	TOTAL SCREENED	REMARKS	ENUMERATION UNIT NUMBER
1	Nigeria International	Old Jeshwang	KMC	WCR-1	10	7	17	05/07/2013	**EU 2**
2	Babylon Day Care & Nursery	Daranka	Kombo North	WCR-1	38	67	105	14/06/2013	**EU 2**
3	Star Preparatory School	Pipeline	KMC	WCR-1	40	34	74	13/05/2013	**EU 2**
4	Nemasu Nursery Madarassa	Sukuta	Kombo North	WCR-1	22	25	47	14/05/2013	**EU 2**
5	Kotu Preparatory	Kotu	KMC	WCR-1	23	25	48	13/05/2013	**EU 2**
6	Marent	Ebo Town	KMC	WCR-1	49	51	100	05/10/2013	**EU 2**
7	Bintang Bolong	Tallinding	KMC	WCR-1	15	20	35	05/02/2013	**EU 2**
8	J.T.T. Gilipiti	Tallinding	KMC	WCR-1	23	19	42	05/06/2013	**EU 2**
9	Clunny Nursery	Banjul	Banjul	WCR-1	48	63	111	05/08/2013	**EU 2**
10	Sobeya	Tallinding	KMC	WCR-1	16	23	39	30/04/2013	**EU 2**
11	Karin International	Bundung	KMC	WCR-1	38	35	73	05/07/2013	**EU 2**
12	Wisdom Nursery	Serekunda	KMC	WCR-1	21	24	45	06/06/2013	**EU 2**
13	Overcomers International	Dippa Kunda	KMC	WCR-1	13	17	30	05/07/2013	**EU 2**
14	Ellis Demaro	Bakoteh	KMC	WCR-1	21	18	39	13/05/2013	**EU 2**
15	D. Harris International	Manjai	KMC	WCR-1	14	14	28	05/07/2013	**EU 2**
16	Jahanka	Jahanka	Fulladu West	**CRR**	21	27	48	19/06/2013	**EU 2**
17	Sofaniama	Katamina	Niamina	**CRR**	26	23	49	18/06/2013	**EU 2**
18	Jahan Annex		Fulladu West	**CRR**	15	38	53	18/06/2013	**EU 2**
19	Kerewan Samba Sira	K. Samba Sira	Lower Saloum	**CRR**	39	58	97	17/06/2013	**EU 2**
20	Sare Luba	Sare Luba	Fulladu West	**CRR**	12	15	27	17/06/2013	**EU 2**
21	Brikama -Ba	Brikamaba	Fulladu West	**CRR**	40	40	80	17/06/2013	**EU 2**
22	Kerewan Dumbokono	Kerewan Dumbo	Fulladu West	**CRR**	8	15	23	18/06/2013	**EU 2**
23	Barajally Suba	Barajally Suba	Niani	**CRR**	29	24	53	17/06/2013	**EU 2**
24	Dodo	Dodo	Sami	**CRR**	17	36	53	17/06/2013	**EU 2**
25	Ballagharr Kerr Ndery	Ballanghar K. Ndery	Lower Saloum	**CRR**	15	38	53	18/06/2013	**EU 2**
26	Mamut Fana	Mamut Fana	Niani	**CRR**	28	42	70	18/06/2013	**EU 2**
27	Kataba Abdou Ndow	Kataba Abdou Ndow	Niani	**CRR**	19	34	53	17/06/2013	**EU 2**
28	Pallol Wollof (Pallang)	Pallol Wollof	Lower Saloum	**CRR**	33	26	59	18/06/2013	**EU 2**
29	Kerr Auldi	Kerr Auldi	Lower Saloum	**CRR**	26	27	53	18/06/2013	**EU 2**
30	Baati Ndarr	Baati Ndarr	Upper Saloum	**CRR**	22	38	60	18/06/2013	**EU 2**
31	St James Nursery School	Siffoe	Kombo South	WCR-2	11	12	23	06/06/2013	**Eu 1**

32	St. Bernadet Nursery	Mayok	Foni Bintang	WCR-2	16	33	49	16/05/2013	**EU 1**
33	Grace Nursery/Kaimo	Kaimo	Foni Bintang	WCR-2	31	29	60	17/05/2013	**EU 1**
34	Katakorr Nursery	Karakorr	Foni Bintang	WCR-2	22	25	47	17/05/2013	**EU 1**
35	Fresh Start Foundation	Brikama Misera	Kombo Central	WCR-2	21	29	50		**EU 1**
36	Aja Asombi Bojang Nursery School	Kanilai	Foni Kansala	WCR-2	30	25	55	16/05/2013	**EU 1**
37	Dumbuto	Dumbuto	Kiang West	LRR	17	18	35	06/06/2013	**EU 1**
38	Jissay (Manduar)	Jissay	Kiang West	LRR	25	18	43	06/05/2013	**EU 1**
39	Tankular	Tankular	Kiang West	LRR	20	12	32	06/05/2013	**EU 1**
40	Massembeh	Massembe	Kiang West	LRR	14	12	26	06/06/2013	**EU 1**
41	Madina Angalleh	Madina Angalleh	Kiang West	LRR	30	28	58	06/06/2013	**EU 1**
42	Nema ABC	Nema	Kiang Central	LRR	60	51	111	06/06/2013	**EU 1**
43	Kerr Ngorr	Kerr Ngorr	Jokadou	NBWR	31	19	50	06/05/2013	**EU 1**
44	Kerr Amadou	Kerr Amadou	Jokadou	NBWR	23	27	50	06/12/2013	**EU 1**
45	Kerr Alhagie Malick	Kerr Alhagie	Jokadou	NBWR	29	35	64	06/04/2013	**EU 1**
46	Daru Fodayba	Daru Fodayba	Jokadou	NBWR	30	23	53	06/12/2013	**EU 1**
47	Aljamdu	Aljambu	U/Niumi	NBWR	22	30	52	06/03/2013	**EU 1**
48	Kerr Mamma	Kerr Mamma	U/Niumi	NBWR	59	23	82	06/03/2013	**EU 1**
49	Saaba	Saaba	L/Badibou	NBER	30	23	53	06/11/2013	**EU 1**
50	Kinteh Kunda Annex	Kinteh Kunda	L/Badibou	NBER	23	30	53	06/10/2013	**EU 1**
51	Banni Nursery Annex	Banni	L/Badibou	NBER	22	31	53	06/11/2013	**EU 1**
52	Sen Jons		U/Badibou	NBER	21	29	50	06/10/2013	**EU 1**
53	Farafenni Proper Nursery Annex	Farafenni	U/Badibou	NBER	19	34	53	06/12/2013	**EU 1**
54	Makka Farafenni Nursery Annex	Farafenni	U/Badibou	NBER	25	25	50	06/10/2013	**EU 1**
55	Khalid Bun Walid (Garawol)	Garawol	Kantora	URR	24	11	35	06/12/2013	**EU 1**
56	Baniko Ismaila	Baniko	Fulladu East	URR	12	30	42	13/06/2013	**EU 1**
57	Sare Alpha (LBS)	Sare Alpha	Kantora	URR	28	24	52	06/12/2013	**EU 1**
58	Nawdeh	Nawdeh	Sandu	URR	15	20	35	06/11/2013	**EU 1**
59	Basse SOS Nursery	Basse	Fulladu East	URR	31	21	52	06/10/2013	**EU 1**
60	Song Kunda	Song Kunda	Kantora	URR	14	9	23	06/12/2013	**EU 1**
61	Makamasereh	Makamasireh	Wulli	URR	13	12	25	06/11/2013	**EU 1**
